# Newspaper framing of food and beverage corporations’ sponsorship of sport: a content analysis

**DOI:** 10.1186/s12889-022-14031-w

**Published:** 2022-09-16

**Authors:** L. E. Carters-White, C. Patterson, A. Nimegeer, S. Hilton, S. Chambers

**Affiliations:** 1 MRC/CSO Social and Public Health Science Unit, University of Glasgow, 99 Berkeley Street, Glasgow, G3 7HR, Berkeley Square, United Kingdom; 2 SPECTRUM Consortium, Usher Institute of Population Health Sciences and Informatics, Doorway 1, Old Medical School, Teviot Place, University of Edinburgh, Edinburgh, United Kingdom EH8 9AG; 3 School of Social and Political Sciences, University of Glasgow, Glasgow, United Kingdom

**Keywords:** Marketing, Food, Framing, Obesity, Sport sponsorship

## Abstract

**Background:**

Unhealthy diets are a leading contributor to obesity, disability and death worldwide. One factor cited as contributing to rises in obesity rates is the pervasive and ubiquitous marketing of unhealthy foods and beverages (F&Bs) across a variety of mediums, such as sport sponsorship at both professional and amateur levels. Despite increased academic attention on the detrimental impacts of sport sponsorship within the obesogenic environment, this has not been matched by legislative action. One explanation may be the way that F&B corporations’ sport sponsorship is framed within policy debates. Framing is the deliberate ways in which (often contested) issues are presented in communication. This paper examines how sport sponsorship by F&B corporations is framed through media reports.

**Methods:**

This study employed a mixed methods content and framing analysis. First, we conducted a quantitative newsprint content analysis (*n* = 234). This then informed and directed a thematic framing analysis of a sub-set of articles (*n* = 54) that specifically associated sport sponsorship by F&B corporations with obesity and childhood obesity.

**Results:**

The findings suggest that two competing frames are evident within newspaper coverage: 1) public health and 2) industry. The public health frame rejects the sponsorship of sport by High in Fat Sugar and Salt (HFSS) product corporations in particular, calling for such sponsorship to be restricted or banned. The industry frame characterises sponsorship of sport as a form of corporate social responsibility, positioning industry as good moral actors and part of the solution to childhood and adult obesity. These frames are evident across other Unhealthy Commodity Industries (UCIs) policy debates. However, the prominence of industry actors within the sample is potentially indicative of their discursive power within this space, particularly with their emphasis on the financial maintenance of sport as well as encouraging physical activity, contributing to the lack of regulatory development of sport sponsorship by F&B corporations.

**Conclusions:**

The findings of this study are particularly useful for public health organisations who seek regulatory change, as it may provide further insight into countering industry framing practices, raising the salience of regulation of sport sponsorship and thus increasing the likelihood of regulatory development that seeks to improve population health.

**Supplementary Information:**

The online version contains supplementary material available at 10.1186/s12889-022-14031-w.

## Background

Non-communicable diseases (NCDs), such as Type 2 diabetes, cancers and respiratory diseases contribute to approximately 41 million deaths per year globally [1]. NCDs have been identified as attributable to almost 89% of deaths in England [2], and unhealthy diets are a leading contributor to obesity and disability [3, 4]. One factor cited as contributing to rises in obesity rates is the pervasive and ubiquitous marketing of unhealthy foods and beverages (F&Bs) across a variety of mediums, such as through digital marketing, price promotions and sport sponsorship at both professional and amateur levels [5]. In recent years, the role of sport sponsorship by F&B corporations in debates about obesity has received increased attention due to the prominent position sport occupies in society [6]. Evidence suggests that sport sponsorship by brands selling products predominantly high in fat, sugar and salt (HFSS) can positively impact perceptions of HFSS corporations, with one study reporting that children perceived HFSS food sponsors as ‘cool’ if they sponsored their favourite sports teams or athletes. Bragg et al.’s [7] recent systematic review found sport sponsorship by F&B corporations often promoted HFSS products, and athlete endorsement of HFSS products promoted mixed messaging around the healthfulness of sport and the consumption of HFSS products. As such, there have been increasing calls, both within the United Kingdom (UK) and globally, for the implementation of comprehensive and effective regulation seeking to limit F&B corporation sponsorship of both amateur and professional sports [1, 8].

Despite increased academic attention on the detrimental impacts of sport sponsorship on dietary preferences and the broader obesogenic environment [8], this has not been matched by legislative action [9, 10]. The UK Government will further regulate HFSS product marketing across television, online and promotional displays in supermarkets in 2022 [11–13], but these regulations will not cover sport sponsorship by F&B corporations, leaving this avenue of marketing largely open to exploitation. However, there is precedent for statutory legislation limiting sport sponsorship by other unhealthy commodity industries (UCIs), notably successful [14] comprehensive bans on tobacco sport sponsorship or advertising in line with the Article 13 of the WHO’s Framework Convention on Tobacco Control (FCTC) [1]. There has also been increased academic scrutiny of sports sponsorship by the gambling and alcohol industries [15–18]. In contrast, and despite growing understanding of the impact of HFSS sponsorship in sports, the issue has made relatively little impact on policy debates.

The sponsorship of sport by F&B corporations occurs in tandem with sport often being presented as a way for both children and adults to lead healthier, more active lifestyles [19, 20]. Ofcom [21] reported that on YouTube, 28% of 5–15 year-olds viewing time consisted of watching sports/football clips or videos. Sports programmes were found to be one of the most popular viewing programmes for children on television [21]. Sport clearly plays an important role within children’s lives.

As such, there appears to be a misalignment between the emerging evidence of the impact of sport sponsorship by HFSS corporations and policy development, raising the question of why this may be. Birkland [22] highlights that policymaking includes decisions not to develop or implement policy. As outlined by Smith [23], often policy development, or the decision not to implement a policy, results from the successful advocacy of ideas by competing interests. Lack of policy development around sports sponsorship therefore may be explained by the way that F&B corporations’ sport sponsorship is positioned or framed within policy debates by various policy actors. The act of framing, as described by Entman (1993, p53), suggests that:

*To frame is to select some aspects of perceived reality and make them more salient in a communicating text, in such a way as to promote a particular problem definition, causal interpretation, moral evaluation, and/or treatment recommendation for the item described.*

As such, the framing of an issue can impact on how readily that issue is taken up within the policy arena, and contributes to a broader process of agenda-setting [24, 25]. The media represent a key influence on perceptions of health issues, setting the public agenda by giving prominence to certain issues and omitting others [26]. Applying frame analysis to representations of issues (in the media, consultations or other dissemination avenues), can therefore present potential explanations for the success of some policy issues or solutions, and for the failure of others to be implemented or even acknowledged [25]. Entman’s [27] definition assumes an intentionality behind the deployment of a frame, and suggests that political actors’ use of discourse within policy debates is driven by a desire to influence policymaking in line with their interests [28]. As such, examining media frames represent a valuable way to understand the framing practices of actors seeking to enact or hinder policy change.

As far as the researchers are aware, no such analysis has been conducted on how the issue of sport sponsorship by F&B corporations has been conducted. This paper aims to examine how sport sponsorship by F&B corporations is framed through media reports. By improving the understanding of how sport sponsorship by F&B corporations is framed within discourse, the public health community may be better able to counteract frames which undermine the need for regulation.

## Methods

This study employed a mixed methods content and framing analysis. First, we conducted a quantitative newsprint content analysis using a method developed by Hilton and colleagues [29–33] to ensure systematic coding of newspaper content (see Additional File 1). This then informed and directed the second stage of the study, where we conducted a thematic framing analysis of a sub-set of articles that specifically associated sport sponsorship by F&B corporations with obesity and childhood obesity (see Additional File 2). We employed Entman’s [27] definition of framing as the theoretical base from which to inform and conduct the study, and this particularly directed the qualitative element.

### Sample selection

We selected 10 UK national newspapers based on highest circulation Figs. [34] to include a range of UK newspaper audiences. However, The Observer, Times/Sunday Times newspapers returned no results, and thus eight UK national newspapers were included in the final sample. We did not select newspapers based on ideological or political spread.

We selected a time period of 1^st^ January 2009 to 1^st^ February 2019. Following similar analyses of trends in newspaper reporting around health [26–30] this ten-year period allowed for an examination of change of coverage over time. In addition, from 2009 there was an increased policy focus on advertising of foods High in Fat, Sugar and Salt to children [35], and this time period included key sporting and policy events such as the 2012 Olympic Games in London and the publication of the UK Government’s Childhood Obesity Plan [36].

### Database search and manual article elimination

We searched the Nexis database combining the following search terms: 1) ‘sport or Olympics or football’, 2) ‘sponsorship or advertising or marketing’ (three or more mentions), and 3) ‘food or McDonald’s or Cadbury’s or Coca Cola’. We assessed articles against the following exclusion criteria: 1) less than 10% of the article about sport sponsorship, 2) a reader’s letter, and 3) a section of a television guide. Initially, less than 50% of the article about sport sponsorship was used as an exclusion criterion, however this produced so few results that we decided to reduce this percentage in order to generate an adequate sample for analysis.

### Quantitative coding and data analysis

We developed a coding framework to systematically record relevant content in each article (see Additional file 1). The coding frame was developed based on Hilton and colleagues’ [32] content analysis methodology and included: basic descriptive characteristics of the articles, the topics covered, actors represented, and actors’ tone towards sport sponsorship (positive, negative, neutral). We tested and refined the coding framework on 10% of articles found (*n* = 25), identifying that sub-sample through selection of every 10^th^ article for further examination. Following the piloting of the coding framework, we developed a descriptor document to ensure consistent coding of further articles.

A combination of quantitative and qualitative approaches was used to validate the coding frame. LCW and AN double-coded a 10% sample, and inter-rater agreement for each code was measured using Cohen’s kappa. Where less than substantial agreement (< 0.6) was identified, code definitions were discussed, and the coding frame and descriptors were adjusted. Following this, the variables describing geographical focus of articles were eliminated. All variables, after coding adjustment, achieved between 0.6 and 1.0 Cohen’s kappa coefficient, considered to represent moderate to substantial agreement [37].

The coding process comprised careful reading of the full text of each article, and the recording of whether the article contained content relevant to each thematic code. LCW entered coded data into a spreadsheet that was imported into SPSS Statistics 26 for analysis.

### Qualitative coding and data analysis

After quantitative coding, a sub-sample of 57 articles (referring to obesity and/or childhood obesity) were extracted from the full sample for further qualitative analysis. We based the initial coding frame around Entman’s [38] concepts of problem definition, causal interpretation, moral evaluation and solutions (replaced treatment recommendations as more suited to research context). Following the principles of the constant comparative method [39, 40], the full sub-sample was analysed against the coding frame, with any new inductively-generated themes added to the coding frame (see Additional file 2). LCW conducted the qualitative coding, with SC double-coding 20%, and any disagreements were discussed until consensus was reached. The study team met regularly to discuss the analysis, work through disagreements, and reach consensus across the findings.

### Results: quantitative content analysis

#### Description of sample

The final analysis included 234 articles. The Guardian (*n* = 76), The Independent (*n* = 40), and The Daily Telegraph (*n* = 37) contained the highest number of articles relating to sport sponsorship by F&B corporations. Articles’ mean word count was 653 words.

**Fig. 1:**
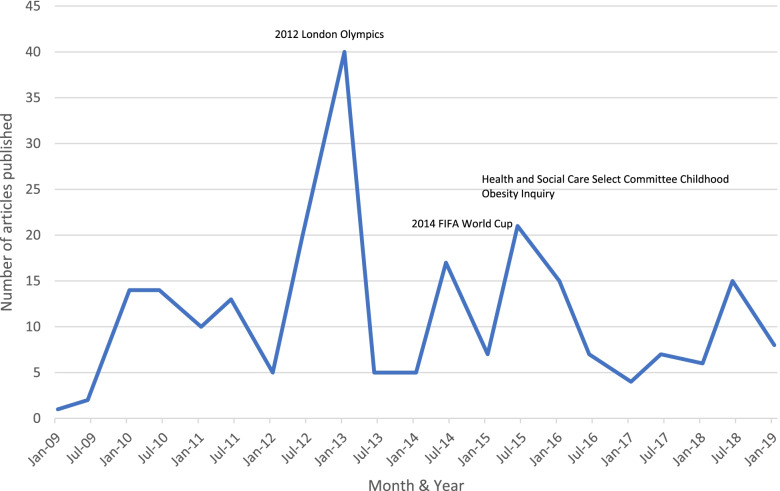
Frequency of articles over time from January 2009 to January 2019

Figure 1. illustrates the distribution of articles over the sample time period, grouped into six-month periods, with three distinct peaks for 2012, 2014 and 2015. These peaks in reporting coincide with three events: 1) the 2012 London Olympics, 2) the 2014 FIFA World Cup, and 3) the Health and Social Care Select Committee’s Childhood Obesity Inquiry.

Adult professional sport was the most frequently referenced type of sport (*n* = 152, 65%). The Olympics was the sporting event referred to most frequently (*n* = 84, 35.7%), followed by football competitions (*n* = 73, 31.2%). The soft drink industry (*n* = 198, 83.8%) and HFSS food industry (*n* = 145, 62%) were the advertisers mentioned most frequently.

**Fig. 2:**
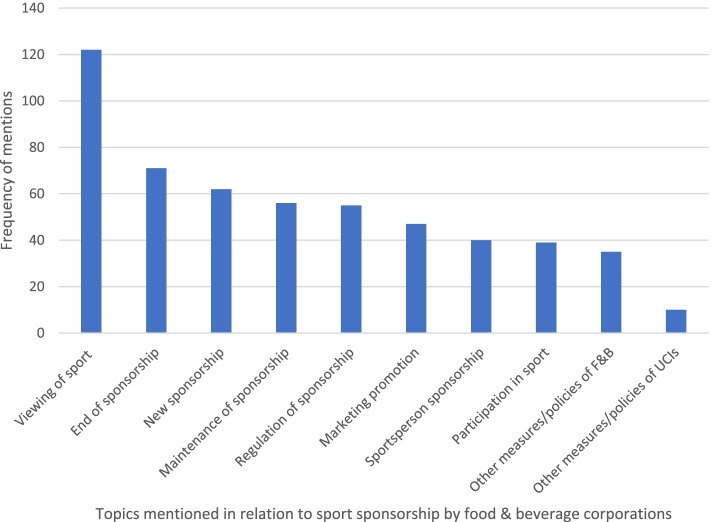
Frequency of topics mentioned in relation to sports sponsorship by F&B corporations

Figure 2. illustrates the distribution of topics that were specifically coded for throughout the quantitative content analysis. The viewing of sport was most frequently referenced (*n* = 123, 52.1%), followed by sports sponsorship contracts ending and/or being cancelled (*n* = 71, 30.3%). Participation in sport was the third least referenced topic (*n* = 39, 16.7%), indicating that in the sample, sport sponsorship by F&B corporations was mostly associated with the consumption of sport rather than participation.

#### Actors and their description of sport sponsorship by F&B corporations

We wanted to understand which categories of actors spoke about sport sponsorship by F&B corporations. Table 1. shows the number of times specific categories of actor were mentioned (either within the body of the article or through included quotes). Although multiple categories of actor were mentioned or spoke within the articles, F&B industry actors were the category mentioned most frequently (*n* = 70, 24%). We coded each actor mentioned for their presented attitude to sport sponsorship, positive, negative or neutral. F&B industry actors, sport representatives, the advertising industry and sportspersons were more likely to be reported as describing sport sponsorship as a positive relationship. In comparison, public health advocates, researchers and politicians and government were reported to more frequently present negative views towards sport sponsorship. Public health advocates included actors from departments of public health, non-governmental organisations and charities. F&B industry actors worked for specific F&B corporations or F&B trade associations. Advertising industry actors included advertisers, advertising trade associations and broadcasters.

**Table 1:** Mentions of actors in sample and tone towards sport sponsorship by F&B corporations

Category of actor	Number of mentions in articles	Percentage (%)	Tone of comments		
			Positive (%)	Negative (%)	Neutral (%)
**F&B Industry**	70	24.22	47.14 (n = 33)	37.14 (n = 26)	15.71 (n = 11)
**Journalists or Commentary**	66	22.83	12.12 (n = 8)	42.42 (n = 28)	45.45 (n = 30)
**Sport Representatives**	51	17.64	74.50 (n = 38)	15.68 (n = 8)	9.80 (n = 5)
**Public health advocates**	43	14.87	2.32 (n = 1)	95.34 (n = 41)	2.32 (n = 1)
**Advertising industry**	21	7.26	57.14 (n = 12)	38.09 (n = 8)	4.76 (n = 1)
**Researchers**	14	5	14.28 (n = 2)	78.57 (n = 11)	7.14 (n = 1)
**Politicians & government**	13	4.84	30.76 (n = 4)	61.53 (n = 8)	7.69 (n = 1)
**Sportspersons**	9	3.11	66.66 (n = 6)	22.22 (n = 2)	11.11 (n = 1)
**Government health organisation**	2	0.69	0 (n = 0)	100 (n = 2)	0 (n = 0)
**Civil servant**	1	0.34	0 (n = 0)	100 (n = 1)	0 (n = 0)
**Government spokesperson**	0	0	0 (n = 0)	0 (n = 0)	0 (n = 0)
**TOTAL**	289	100	NA	NA	NA

**Fig. 3:**
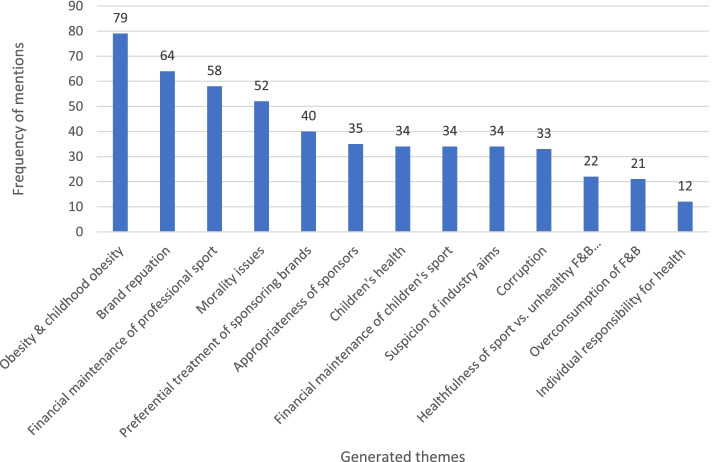
Frequency of generated themes associated with sport sponsorship by food & beverage corporations

The quantitative coding captured different themes that were present in the articles (see Fig. 3). Obesity and childhood obesity was a core theme that was generated during the analysis (*n* = 79), followed by brand reputation (*n* = 64) and financial maintenance of professional sport (*n* = 58). Furthermore, as can be seen in Fig. 1, there were two peaks in media coverage during the London 2012 Olympics as well as the UK Government Health and Social Care Select Committee Inquiry on Childhood Obesity. Analysis demonstrated that media coverage during these time periods centred on concerns over obesity and childhood obesity in the UK, and the study team decided to conduct a further qualitative framing analysis of the sport sponsorship by F&B corporations, obesity and childhood obesity to further unpack the framing employed.

### Results: qualitative framing analysis

The 57 articles that were coded as including obesity & childhood obesity as a theme were subjected to qualitative analysis. Two distinct frames were generated through the data analysis: 1) public health framing, and 2) industry framing. Each of these frames presented different problem definitions, causal interpretations, moral evaluations and solutions, as identified in Entman’s [27] concept of framing.

#### Problem definition

The public health frame typically defined the problem of childhood obesity or adult obesity by its scale, suggesting that childhood obesity or adult obesity were public health problems requiring immediate policy attention:

*The campaigners warn that child obesity has reached "epidemic" proportions and that the country is facing a "public health crisis"...* (The Daily Telegraph, Article 21)

As well as scale, childhood obesity was positioned as a complex crisis, with much emphasis placed on the need for public health to ‘battle’ such a crisis.

Within the public health frame, obesity was described as a health and financial burden:

*Government healthy eating campaigns can't possibly compete, so it's not surprising that the cost of obesity to people's health, the NHS and wider society, is spiralling out of control.* (The Daily Mail, Article 5)

In addition, public health frames tended to primarily define children as an affected group when considering both sport sponsorship and childhood obesity, with secondary focuses on young people, parents, and adults. There was a specific focus on those with low socioeconomic status, who were positioned as particularly vulnerable to childhood obesity:

*Obesity rates are highest for children from the most disadvantaged communities...* (The Express, Article 1)

Industry frames provided no definition of the problem of childhood or adult obesity when they discussed sport sponsorship. Arguably, this could be interpreted as industry frames deliberately downplaying any relationship between obesity and sport sponsorship.

#### Causal interpretation

Public health frames suggested that sponsorship of sport by F&B corporations contributes to childhood obesity and adult obesity. These frames tended to suggest that sponsorship of sport by brands typically associated with HFSS products created an association between the healthy activity of sport and unhealthy food and soft drinks:

*The letter... accuses food companies of triggering a "halo effect" around unhealthy foods by associating them with sport.* (The Independent, Article 22)

This health halo was framed as potentially contributing to poor health by encouraging consumption of HFSS products. This was considered particularly problematic due to the impact this form of marketing may have on children, who were framed as vulnerable to such marketing practices. In addition, sport sponsorship by F&B corporations was contended to be a marketing technique to evade regulations intended to protect children’s health:

*Caroline Cerny… suggested junk food firms are using the sponsorship of sport to circumvent rules preventing them from using advertising to target children.* (The Daily Mail, Article 1)

Industry framing was at odds with the assertion that sponsorship of sport by F&B corporations encouraged poor dietary preferences contributing to obesity. Rather, they contended that sport sponsorship may encourage physical activity and thus combat obesity:

*One of its [McDonald’s] executives even argued recently that its initiatives offer youngsters a chance to burn off excess calories accumulated by eating junk food.* (The Daily Mail, Article 19)

In addition, industry actors occasionally referred to the evidence base to legitimise their argument:

*Ian Barber, the director of communications at the Advertising Association, said: "Much analysis has been done about advertising's link with our obesity problem. Prof David Buckingham - an independent expert - sums it up when he says, 'the impact, if any, is small'."* (The Guardian, Article 50)

Through industry’s minimal acknowledgement of obesity or childhood obesity, plus their categorisation of sport sponsorship as not a contributing factor of poor dietary preferences and obesity, it appeared industry actors were constructing frames intended to diminish the persuasiveness of public health framing.

#### Moral evaluation

Public health framing and industry framing differed most starkly in their moral evaluations of sport sponsorship. Public health framing tended to cite the protection of children’s health as a reason to reduce sport sponsorship by F&B corporations:

*This continued failure to protect children from this type of marketing not only represents extremely poor judgement, but a dereliction of public health responsibilities.* (The Independent, Article 12)

In addition, public health framing expressed scepticism as to industry’s aims when sponsoring sport:

*The marketing, availability and price of unhealthy food all influence individual decisions about what to eat and when to eat it. Junk food companies sponsoring kids' sports out of the goodness of their hearts? What a lovely thought.* (The Guardian, Article 18)

This scepticism was often related to a perception that food and drink industry sport sponsorship was conducted to create *“social value”* for the brand, improving their overall brand reputation and fostering positive attitudes. This was often particularly associated with HFSS product brand sponsorship of the Olympics in 2012 in London.

In contrast, industry framing cited protection of industry and protection of sport as a reason to not reduce F&B sponsorship of sport:

*McDonalds reacted to concerns about involvement with the Olympics by insisting sponsorship was essential to the success of the Games.* (The Guardian, Article 42)

By citing that sponsorship protected sport, it appeared that industry framing attempted to position these sponsoring brands as ‘good’ moral actors. This protection was often mentioned when referencing the funding of children’s sport, suggesting that brands are responsible for ensuring children’s access to sport and physical activity.

#### Solutions

Public health framing overwhelmingly supported a restriction or ban on sport sponsorship by HFSS brands. They occasionally cited other unhealthy commodities which were regulated through similar solutions:

*Their recommendations include…a ban on sponsorship of sports clubs and youth tournaments by brands associated with unhealthy products. They are proposing, in other words, to treat unhealthy food pretty much like tobacco.* (The Daily Telegraph, Article 1)

Public health frames were also critical of existing policy, citing it as ineffective in protecting children’s health from marketing of HFSS products, particularly in sport settings. They were sceptical of industry as being part of the solution to childhood obesity, often arguing against industry claims that their sponsorship of sport increased uptake of physical activity.

Industry framing cited policy solutions that focused on individual behaviours, such as encouraging healthy eating practices and increasing physical activity amongst children:

*Professor Gately… said… the McDonald's campaign should be embraced because it provided a route to large numbers of people. "Without public-private partnership we are not going to address these issues about the promotion of physical activity or healthy eating or tackling obesity".* (The Guardian, Article 59)

Industry framing maintained that current regulatory policies for marketing HFSS products were effective, and that the financing of sport through sponsorship was essential. They rejected the banning of sport sponsorship:

*Kent says the criticisms miss the point. If it wasn't for the major sponsors putting tens of millions of pounds into the Olympics every year (Coke refuses to say how much), the Games would not exist in their present form.* (The Sunday Telegraph, Article 5)

## Discussion

This study is important in understanding potential links between sport sponsorship by F&B corporations and childhood obesity. To date, generating evidence on this link has been slow and as a result regulation has also been slow [10]. Our findings indicate that the sponsorship of sport by F&B corporations is not a prominent news item; low media prominence of the topic may hinder traction of the topic on the political agenda and its potential solutions within policy discourse, despite public health advocacy. While sponsorship of sport by F&B corporations was discussed relatively infrequently in UK newspapers, what coverage there was often focused on its relationship with obesity and childhood obesity, which could be advantageous to public health policy advocacy. However, the findings also suggest that sport sponsorship is a mechanism through which F&B corporations demonstrate their ability to add social value to their brands as part of their broader corporate social responsibility (CSR) activities, which may harm the public health case for regulation.

Overall, newspaper discussions of sport sponsorship by F&B corporations and obesity and/or childhood obesity were often presented through either a public health frame or industry frame. These frames aligned with broader frames commonly referred to as social and market justice, which have been evidenced across other UCI debates [25, 41–43]. Social justice frames centre on values of equity, health for all, and protection of the vulnerable [41]. Market justice frames, in contrast, tend to centre on values of individual responsibility for health, protection of capitalist endeavours, and rejection of state intervention [41]. Within this study it was evident that although the public health frame sought to define sports sponsorship as problematic in relation to childhood obesity and obesity, industry framing omitted reference to this issue. In fact, industry actors were explicit in their denial of sport sponsorship by F&B corporations as problematic.

An important finding of the study was it appeared that part of industry framing was to emphasise sport sponsorship by F&B corporations as a social good, through the financing and maintenance of sport (at both professional and amateur level), thus being a potential solution to the obesity problem. Industry framing emphasised the role of industry as part of the solution, arguably as an attempt to legitimise sport sponsorship and ensure that regulation, as was being recommended within public health framing, was not viewed as fair or necessary. As such, it could be argued that industry framing was attempting to position F&B corporations as good corporate citizens, defined by Dorfman et al. [44] as when companies aim to emphasise their role in bettering society as a means to override the potential harm their products or services may cause. This framing also created an ‘illusion of righteousness’, whereby industry portrayed themselves as morally and socially good to deter or undermine attempts to regulate their business practices [25, 45].

A further important finding of the study was how children were positioned between a moral battlefield of public health versus industry framing. Public health frames were primarily focused on the protection of children’s health, with children designated as a vulnerable population in need of protection from harmful industry marketing practices. Similar ‘protection’ frames were seen in Hilton et al.’s [46] study, where it was argued that there exists a duty to protect children from harms. In contrast, industry frames often highlighted the benefit that sport sponsorship brought to children through the financial maintenance of sport. Once again, this frame feeds into a wider narrative that industry provides a social good, and that regulation of sport sponsorship would be a social wrong that would detrimentally impact both children’s health and social wellbeing. The tension between the two overarching public health and industry frames of how children are best protected is potentially indicative of the role that each set of actors’ view themselves as playing in the debate: who should and is best to protect children and their health? It arguably speaks to broader debates on who should be considered legitimate actors within policy debates, and how these actors employ framing to enhance their own legitimacy whilst undermining others [24].

The overarching finding of the study is that an increasing emphasis on the need to regulate sport sponsorship by F&B corporations within academic literature and governmental healthy weight strategy documents [8, 10] does not appear to have made an impact on mass media discourse. Given the influence that media content exerts on public and political attention and understandings, this lack of impact may partially explain why regulation of sport sponsorship by F&B corporations has seen little policymaking progress.

Although there was a public health frame generated through the analysis, it was not the dominant framing identified. Industry actors were the most prominent actors represented in the sample, and while the themes of obesity and childhood obesity were prominent, the predominance of the industry framing suggests that sports sponsorship has not been adequately problematized within media discourse, and potentially illustrates the power of industry actors to influence discourse [23, 24]. Despite the aligning of frames with other UCI debates [43, 47–49], which is suggestive of sport sponsorship by F&B corporations being viewed as a similar marketing technique to other forms of marketing, such as television or online advertising, the relatively low media presence of public health voices within media discourse may have contributed to the lack of regulatory attention it has received.

### Limitations

A limitation of this study was the small sample size of articles that met the inclusion and exclusion criteria. However, combined quantitative and qualitative analysis enabled an in-depth analysis of the sample, which mitigates its limited sample size. The national focus of the newspaper articles in this study, and the transnational corporations searched for, meant there was a limited focus on more local sponsorship arrangements. Sponsorship of children’s sport in the UK is an area that requires further academic attention. A further potential limitation is that although this study recognises the importance of online sources as part of the broader media landscape, as well as the increasing inclusion of it in framing analysis, traditional media outlets such as print newspapers remain influential [50]. The online version of print newspapers largely dominate online news readership in the UK, and arguably define or legitimise news agendas in online sources or discussions [26]. As such, traditional print media such as newspapers remain an integral part of the broader news landscape, and therefore a relevant subject for analysis, particularly when examining how policy issues are discussed or framed.

## Conclusion

Despite increased attention in the academic literature [1, 6, 7] and by public health organisations [8, 10] about the potential harms of sport sponsorship of F&B corporations, this is not reflected in recent news media discourse. The findings suggest that two frames are evident within newspaper coverage: 1) public health and 2) industry. The public health frame rejects the sponsorship of sport by HFSS product corporations in particular, calling for such sponsorship to be restricted or banned. The industry frame characterises sponsorship of sport as a form of corporate social responsibility, positioning industry as good moral actors and part of the solution to childhood and adult obesity. These frames are evident across other UCI policy debates and children. However, the prominence of industry actors within the sample is potentially indicative of their discursive power within this space, particularly with their emphasis on the financial maintenance of sport as well as encouraging physical activity, contributing to the lack of regulatory development of sport sponsorship by F&B corporations. As such, the findings of this study are particularly useful for public health organisations who seek regulatory change, as it is may provide further insight into countering industry framing practices [42], raising the salience of regulation of sport sponsorship and thus increasing the likelihood of regulatory development that seeks to improve population health.

## Supplementary Information

**Additional file 1.** Coding Sheet Sport Sponsorship by Food and Drink Companies.

**Additional file 2.** Qualitative Coding Framework.

## Data Availability

The datasets used and/or analysed during the current study are available from the corresponding author on reasonable request.

## References

[1] Ireland R, Bunn C, Reith G, Philpott M, Capewell S, Boyland E (2019). Commercial Determinants of Health: Advertising of Alcohol and Unhealthy Foods during Sporting Events. Bull World Health Organ.

[2] Office for Health Improvement & Disparities. Preventing illness and improving health for all: A review of the NHS Health Check programme and recommendations. Annex C: Data on the distribution, determinants and burden of non-communicable diseases in England 2021 [Available from: https://www.gov.uk/government/publications/nhs-health-check-programme-review/annex-c-data-on-the-distribution-determinants-and-burden-of-non-communicable-diseases-in-england#:~:text=They%20can%20often%20be%20chronic,to%20NCDs%20%5Bfootnote%204%5D.

[3] Afshin A, Sur PJ, Fay KA, Cornaby L, Ferrara G, Salama JS (2019). Health effects of dietary risks in 195 countries, 1990–2017: a systematic analysis for the global burden of disease study 2017. The Lancet.

[4] Stuckler D, McKee M, Ebrahim S, Basu S (2012). Manufacturing epidemics: the role of global producers in increased consumption of unhealthy commodities including processed foods, alcohol, and tobacco. PLoS Med.

[5] Cairns G, Angus K, Hastings G, Caraher M (2013). Systematic reviews of the evidence on the nature, extent and effects of food marketing to children. A Retrospective Summ Appetite.

[6] Ireland R, Chambers S, Bunn C (2019). Exploring the relationship between big food corporations and professional sports clubs: a scoping review. Public Health Nutr.

[7] Bragg MA, Roberto CA, Harris JL, Brownell KD, Elbel B (2018). Marketing food and beverages to youth through sports. J Adolesc Health.

[8] Obesity Health Alliance. Turning the Tide: A 10-year Healthy Weight Strategy 2021. [Available from: https://obesityhealthalliance.org.uk/turning-the-tide-strategy/].

[9] Sustain. Parents' perspectives of less healthy food and drink through sport 2021 [Available from: https://www.sustainweb.org/news/jun21-summerofsport/].

[10] Bradshaw B, Crowther B, Viggars M. Kicking out junk food: Sports sponsorship and a better deal for health. 2021. [Available from https://www.sustainweb.org/publications/nov21-kicking-out-junk-food/].

[11] UK Government. Tackling obesity: empowering adults and children to live healthier lives 2020 [Available from: https://www.gov.uk/government/publications/tackling-obesity-government-strategy/tackling-obesity-empowering-adults-and-children-to-live-healthier-lives].

[12] UK Government. Restricting promotions of products high in fat, sugar and salt by location and by price: government response to public consultation 2020 [Available from: https://www.gov.uk/government/consultations/restricting-promotions-of-food-and-drink-that-is-high-in-fat-sugar-and-salt/outcome/restricting-promotions-of-products-high-in-fat-sugar-and-salt-by-location-and-by-price-government-response-to-public-consultation#outcome-and-next-steps].

[13] UK Government. Introducing a total online advertising restriction for products high in fat, sugar and salt (HFSS) 2021 [Available from: https://www.gov.uk/government/consultations/total-restriction-of-online-advertising-for-products-high-in-fat-sugar-and-salt-hfss/introducing-a-total-online-advertising-restriction-for-products-high-in-fat-sugar-and-salt-hfss.

[14] Ford ES, Capewell S (2011). Proportion of the decline in cardiovascular mortality disease due to prevention versus treatment: public health versus clinical care. Annu Rev Public Health.

[15] Bunn C, Ireland R, Minton J, Holman D, Philpott M, Chambers S. Shirt sponsorship by gambling companies in the English and Scottish Premier Leagues: global reach and public health concerns. Soccer & Society. 2018:1–12. 10.1080/14660970.2018.1425682 PMC6795541 31619942

[16] Bestman A, Thomas SL, Randle M, Thomas SDM (2015). Children's Implicit Recall of Junk Food, Alcohol and Gambling Sponsorship in Australian Sport. BMC Public Health.

[17] Danson A (2010). Sponsorship by gambling companies in the uk and europe: the opportunities and challenges. J Sponsorship.

[18] Gee S, Thompson A-J, Batty RJ, Rules of Engagement: Sport Sponsorship, Anti-Ambush Marketing Legislation, and Alcohol Images during the, (2011). Rugby World Cup. J Glob Sport Manage.

[19] Eime RM, Young JA, Harvey JT, Charity MJ, Payne WR (2013). A systematic review of the psychological and social benefits of participation in sport for children and adolescents: informing development of a conceptual model of health through sport. Int J Behav Nutr Phys Act.

[20] Telama R, Yang X, Leskinen E, Kankaanpää A, Hirvensalo M, Tammelin T (2014). Tracking of physical activity from early childhood through youth into adulthood. Med Sci Sports Exerc.

[21] Ofcom. Children and Parents: Media Use and Attitudes Report. UK: Ofcom; 2017. [Available from: https://www.ofcom.org.uk/research-and-data/media-literacy-research/childrens/children-parents-2017].

[22] Birkland TA (2015). An introduction to the policy process: Theories, concepts, and models of public policy making.

[23] Smith K (2013). Beyond evidence based policy in public health: The interplay of ideas.

[24] Fuchs D, Lederer MML (2007). The Power of Business. Bus Polit.

[25] Carters-White L, Chambers S, Skivington K, Hilton S (2021). Whose rights deserve protection? framing analysis of responses to the 2016 committee of advertising practice consultation on the non-broadcast advertising of foods and soft drinks to children. Food Policy.

[26] McCombs M. Setting the agenda: Mass media and public opinion: John Wiley & Sons; 2018.

[27] Entman RM. Framing: Toward clarification of a fractured paradigm. J Commun. 1993;43(4).

[28] Coburn CE (2006). Framing the problem of reading instruction: using frame analysis to uncover the microprocesses of policy implementation. Am Educ Res J.

[29] Buckton CH, Patterson C, Hyseni L, Katikireddi SV, Lloyd-Williams F, Elliott-Green A (2018). The palatability of sugar-sweetened beverage taxation: a content analysis of newspaper coverage of the uk sugar debate. PLoS One.

[30] Patterson C, Hilton S, Weishaar H (2016). Who thinks what about e-cigarette regulation? a content analysis of uk newspapers. Addiction.

[31] Patterson C, Semple S, Wood K, Duffy S, Hilton S (2015). A Quantitative content analysis of uk newsprint coverage of proposed legislation to prohibit smoking in private vehicles carrying children. BMC Public Health.

[32] Hilton S, Patterson C, Teyhan A (2012). Escalating coverage of obesity in uk newspapers: the evolution and framing of the “Obesity Epidemic” From 1996 to 2010. Obesity.

[33] Hilton S, Hunt K, Langan M, Bedford H, Petticrew M (2010). Newsprint media representations of the introduction of the hpv vaccination programme for cervical cancer prevention in the UK (2005–2008). Soc Sci Med.

[34] National Readership Survey. NRS Readership Estimaes - Newspapers and Supplements Jan-Dec 2016 2016 [Available from: https://www.nrs.co.uk/downloads/pdf/newspapers_201612.pdf].

[35] Ofcom. Changes in the nature and balance of television food advertising to children: A review of HFSS advertising restrictions. 2008. [Available from: https://www.ofcom.org.uk/__data/assets/pdf_file/0028/23977/hfssdec08.pdf].

[36] HM Government. Childhood Obesity: A Plan for Action. United Kingdom. 2016. [Available from: https://www.gov.uk/government/publications/childhood-obesity-a-plan-for-action].

[37] Landis JR, Koch GG (1977). The measurement of observer agreement for categorical data. Biometrics.

[38] Entman RM (1993). Framing: Toward Clarification of a Fractured Paradigm. J Commun.

[39] Lincoln YS, Guba EG (1985). Naturalistic Inquiry.

[40] Glaser BG, Strauss AL (1967). The Discovery of Grounded Theory.

[41] Beauchamp DE (1976). Public health as social justice. Inquiry.

[42] Dorfman L, Wallack L, Woodruff K (2005). More than a message: framing public health advocacy to change corporate practices. Health Educ Behav.

[43] Jenkin GL, Signal L, Thomson G (2011). Framing obesity: the framing contest between industry and public health at the new zealand inquiry into obesity. Obes Rev.

[44] Dorfman L, Cheyne A, Friedman LC, Wadud A, Gottlieb (2012). Soda and Tobacco Industry Corporate Social Responsibility Campaigns: How Do They Compare?. PLoS Med.

[45] Yoon S, Lam T-H (2013). The illusion of righteousness: corporate social responsibility practices of the alcohol industry. BMC Public Health.

[46] Hilton S, Wood K, Bain J, Patterson C, Duffy S, Semple S (2014). Newsprint coverage of smoking in cars carrying children: a case study of public and scientific opinion driving the policy debate. BMC Public Health.

[47] Hawkins KW, Linvill DL (2010). Public health framing of news regarding childhood obesity in the united states. Health Commun.

[48] Russell C, Lawrence M, Cullerton K, Baker P. The political construction of public health nutrition problems: A framing analysis of parliamentary debates on junk-food marketing to children in Australia. Public Health Nutrition. 2020:1–12. 10.1017/S1368980019003628 PMC10200508 31948503

[49] Hawkins B, Holden C (2013). Framing the alcohol policy debate: industry actors and the regulation of the uk beverage alcohol market. Critical Policy Studies.

[50] Tobitt C. National newspaper ABCs: Industry-wide circulation decline continues as Metro and Sun top the table 2018 [Available from: https://www.pressgazette.co.uk/the-sun-overtakes-mail-online-to-become-uks-biggest-online-newspaper-brand-latest-comscore-data-shows/].

