# Supplementary material for: Newspaper framing of food and beverage corporations’ sponsorship of sport: a content analysis
Source: BMC Public Health. 2022 Sep 16;22:1753. doi: 10.1186/s12889-022-14031-w (PMC9479402; doi:10.1186/s12889-022-14031-w)
Supplement: Supplementary file 1 — Additional file 1. Coding Sheet Sport Sponsorship by Food and Drink Companies. [file 12889_2022_14031_MOESM1_ESM.doc]

# Coding Sheet

| 1  **Article ID** | | | | | |
| --- | --- | --- | --- | --- | --- |
| LETTER | LETTER | NUMBER | NUMBER | NUMBER | NUMBER |

# Sport Sponsorship by Food and Drink Companies

| 2 | **Headline** |
| --- | --- |
|  | |
|  | |
|  | |

| 3 **Publication** |  |
| --- | --- |
| Guardian | 1 |
| Independent / Independent on Sunday | 2 |
| Daily Telegraph / Sunday Telegraph | 3 |
| Daily Mail / Mail on Sunday | 4 |
| Express/ Sunday Express | 5 |
| Mirror / Sunday Mirror | 6 |
| The Sun | 7 |
| The News of the World | 8 |

| 4 **Date** | | | | | |
| --- | --- | --- | --- | --- | --- |
| Day Month Year | | | | | |
|  |  |  |  |  |  |

| 5 **Front page (p1?)** | |
| --- | --- |
| Yes | 1 |
| No | 2 |

| 6 **Word Count** | | | |
| --- | --- | --- | --- |
| NUMBER | NUMBER | NUMBER | NUMBER |

| **Is the article mainly about sport sponsorship by F&B corporations ?** | YES | NO |
| --- | --- | --- |

| **Categories of sport** | **Yes** | **No** |
| --- | --- | --- |
| Football | 1 | 0 |
| Rugby | 1 | 0 |
| Athletics | 1 | 0 |
| Tennis | 1 | 0 |
| Olympics | 1 | 0 |
| Motor racing | 1 | 0 |
| Sport in general | 1 | 0 |

| **Level of sport** | **Yes** | **No** |
| --- | --- | --- |
| Children (amateur) | 1 | 0 |
| Children (professional) |  |  |
| Adult (amateur) | 1 | 0 |
| Adult (professional) | 1 | 0 |

| **Categories of sport competitions** | **Yes** | **No** |
| --- | --- | --- |
| World Cup | 1 | 0 |
| Premier League | 1 | 0 |
| Carabou (FNA Carling) Cup | 1 | 0 |
| FIFA | 1 | 0 |
| Olympics | 1 | 0 |
| Wimbledon | 1 | 0 |
| Six Nations | 1 | 0 |

| **Types of industry/corporation** | **Yes** | **No** |
| --- | --- | --- |
| Non-HFSS food | 1 | 0 |
| Non-HFSS beverages | 1 | 0 |
| HFSS food | 1 | 0 |
| HFSS beverages | 1 | 0 |
| Sellers of F&B | 1 | 0 |
| Advertising representatives | 1 | 0 |
| Food and beverage representatives | 1 | 0 |

| **Country of origin** | **Yes** | **No** |
| --- | --- | --- |
| UK | 1 | 0 |
| Europe | 1 | 0 |
| Worldwide | 1 | 0 |

| **Does the article discuss:** | **Yes** | **No** |
| --- | --- | --- |
| Viewing of sport | 1 | 0 |
| Participation in sport | 1 | 0 |
| New sponsorship | 1 | 0 |
| Maintenance of sponsorship | 1 | 0 |
| End of sponsorship | 1 | 0 |
| Marketing, promotion (not sponsorship) | 1 | 0 |
| Sportsperson sponsorship | 1 | 0 |
| Regulation of sport sponsorship | 1 | 0 |
| Other measures or policies related to F&B | 1 | 0 |
| Other measures or policies related to other UCIs | 1 | 0 |

| **Actors** | **Who is speaking?** | **Do they describe practice of sport sponsorship by F&B companies:** | | | |  | |  | | | | | | | |
| --- | --- | --- | --- | --- | --- | --- | --- | --- | --- | --- | --- | --- | --- | --- | --- |
|  |  | **positively** | **neutrally** | **negatively** | | **Obesity** | **Childhood obesity** | | **Children’s health** | **Healthfulness of sport vs. unhealthy F&B advertising** | **Overconsumption of F&B** | **Individual responsibility for health and consumption** | **Financial maintenance of professional sport** | **Financial maintenance children’s sport** | **Corruption** |
| F&B industry |  |  |  | |  |  |  | |  |  |  |  |  |  |  |
| Advertising industry |  |  |  | |  |  |  | |  |  |  |  |  |  |  |
| Public health advocates |  |  |  | |  |  |  | |  |  |  |  |  |  |  |
| Researchers |  |  |  | |  |  |  | |  |  |  |  |  |  |  |
| Policymakers |  |  |  | |  |  |  | |  |  |  |  |  |  |  |
| Government spokesperson |  |  |  | |  |  |  | |  |  |  |  |  |  |  |
| Civil servants |  |  |  | |  |  |  | |  |  |  |  |  |  |  |
| Government health organisation |  |  |  | |  |  |  | |  |  |  |  |  |  |  |
| Journalists/commentary |  |  |  | |  |  |  | |  |  |  |  |  |  |  |
| Sport representatives |  |  |  | |  |  |  | |  |  |  |  |  |  |  |
| Sportspeople |  |  |  | |  |  |  | |  |  |  |  |  |  |  |

| **Morality issues** | **Appropriateness of sponsors** | **Brand reputation** | **Preferential treatment of sponsoring brands** | **Suspicion regarding F&B industry aims of sponsorship** |
| --- | --- | --- | --- | --- |
|  |  |  |  |  |
|  |  |  |  |  |
|  |  |  |  |  |
|  |  |  |  |  |
|  |  |  |  |  |
|  |  |  |  |  |
|  |  |  |  |  |
|  |  |  |  |  |
|  |  |  |  |  |

| **Sport sponsorship by F&B companies Qualitative commentary** |
| --- |
|  |
