# Supplementary material for: Newspaper framing of food and beverage corporations’ sponsorship of sport: a content analysis
Source: BMC Public Health. 2022 Sep 16;22:1753. doi: 10.1186/s12889-022-14031-w (PMC9479402; doi:10.1186/s12889-022-14031-w)
Supplement: Supplementary file 2 — Additional file 2. Qualitative Coding Framework. [file 12889_2022_14031_MOESM2_ESM.docx]

**Qualitative Coding Framework**

**Research question:**

How is sport sponsorship by food and beverage corporations framed in relation to obesity and childhood obesity in UK newspapers between 2009 and 2019?

(only code for those sections of text that speak to the above)

| **Signature rhetorical devices – Overarching codes** | **Key aspects** | **Prompts** | **Sub-codes & notes** |
| --- | --- | --- | --- |
| Problem definition | Overall description | How is issue described?  What is the emphasis?  Why is the issue a problem? |  |
|  | Type of problem | What type of problem is it? |  |
|  | Affected groups | Who is the issue a problem for? |  |
| Causal interpretation | Main cause | What is identified as the main cause?  Is the cause environmental or individual?  Who/what is to blame for the problem? |  |
|  | Non-causes | What are dismissed or explicitly identified as non-causes? |  |
| Moral evaluation | Core values or principles | What values or principles are evident in the problem representation |  |
| Recommended policy solutions (treatment recommendation) | Policy prescriptions | What solutions are proposed/emphasised?  What issues are included?  Are the solutions targeted or universal?  Who is responsible? |  |
|  | Non-solutions | What issues are excluded?  What solutions are opposed? |  |
|  | Existing policy | What are the views on current policy? |  |
